# Patella Tendon Injuries Secondary to Cement Spacers Used at First-Stage Revision of Infected Total Knee Replacement

**DOI:** 10.3389/fsurg.2015.00011

**Published:** 2015-04-07

**Authors:** Katherine Wilson, Rahul Kothwal, Wasim S. Khan, Rhodri Williams, Rhidian Morgan-Jones

**Affiliations:** ^1^Cardiff and Vale Orthopaedic Centre, Llandough University Hospital, Cardiff and Vale NHS Trust, Cardiff, UK

**Keywords:** patella tendon, total knee arthroplasty, cement spacer, knee revision, wound healing

## Abstract

We describe a series of three patients who sustained patella tendon injuries in infected total knee arthroplasties following the use of a static cement spacer at first-stage knee revision. The patella tendon injuries resulted in significant compromise to wound healing and knee stability requiring multiple surgeries. The mid-term function was poor with an Oxford score at 24 months ranging from 12 to 20. Based on our experience, we advise caution in the use of static cement spacer blocks. If they are to be used, we recommend that they should be keyed in the bone to prevent patella tendon injuries.

## Introduction

Total knee arthroplasty (TKA) is a highly successful procedure that significantly improves the quality of life of patients (1). Deep infection, however, is a disastrous complication following TKA and is very difficult to treat successfully (2). Although a case for one-stage revision has been put forth recently (3), a two-stage procedure described by Insall in 1983 remains the current gold standard in the management of this complex problem (4). At the first-stage, after explantation of the infected prostheses and cement, and debridement of the infected tissues, antibiotic-impregnated cement spacers are implanted. The patient is given parenteral antibiotics for approximately 6 weeks. Re-implantation of the definitive prosthesis is done after an interval of 6 weeks or more when there is clinical and hematological evidence of the infection having resolved.

Two types of cement spacers are commonly used in clinical practice. These are either “static” or “articulating” cement spacers (5). Advocates of cement spacers state that they deliver high doses of antibiotics locally, increase patient comfort, allow mobility, and provide joint stability. They also minimize contracture of collateral ligaments, thereby facilitating re-implantation of a definitive prosthesis at a later stage (6). The use of these cement spacers, however, is not without complications. Complications that have been described in the literature in relation to their use are spacer related bone loss, instability, implant extrusion, overstuffing, extensor mechanism shortening, spacer fracture, peri-prosthetic fracture, capsular contracture, and difficult subsequent exposure (5). Although mobile spacers have theoretical advantages of maintaining some range of movement, there is no evidence in the literature to suggest their superiority over static spacers.

The purpose of this case series is to report a major complication of patella tendon ruptures that occurred secondary to the use of static cement spacer blocks in a series of three patients undergoing two-stage revision TKA for infection. Based on our experience in dealing with this complex problem, we would like to make suggestions as to how to avoid this complication.

## Case Series

The senior author (Rhidian Morgan-Jones) has a tertiary referral practice for infected TKAs. We describe three patients referred to the senior author from other hospitals since 2004 with patella tendon ruptures secondary to static cement spacers inserted for deep infected TKAs following the first-stage revision operation performed at the referring hospital. Institutional approval and patient consent for inclusion of their case in publication and use of their radiographic images was obtained.

### Case 1

A 73-year-old male underwent a primary right TKA for osteoarthritis that was complicated by a deep infection diagnosed 14 months later. The infected prosthesis was removed, the knee was debrided, and a static cement spacer disk was inserted in the joint space at the referring hospital.

**Figure 1:**
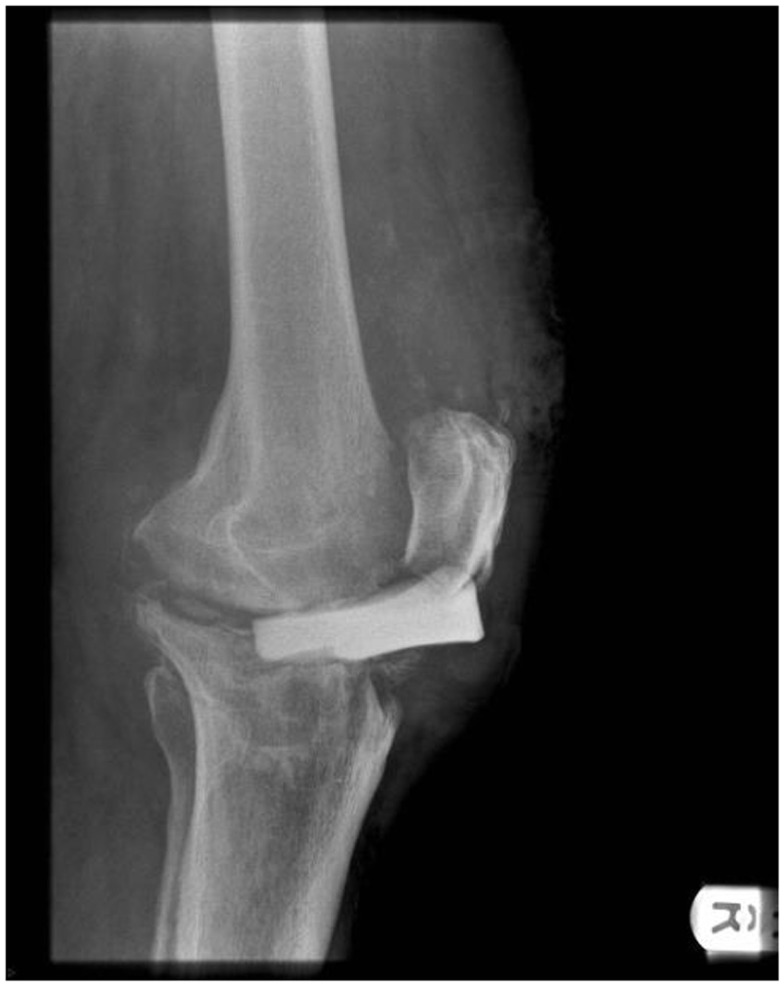
**Complete patella tendon rupture in Case 1 demonstrated by the lateral radiographs of the right knee showing significant anterior displacement of the static cement spacer block**. Note that the block was not keyed in.

**Figure 2:**
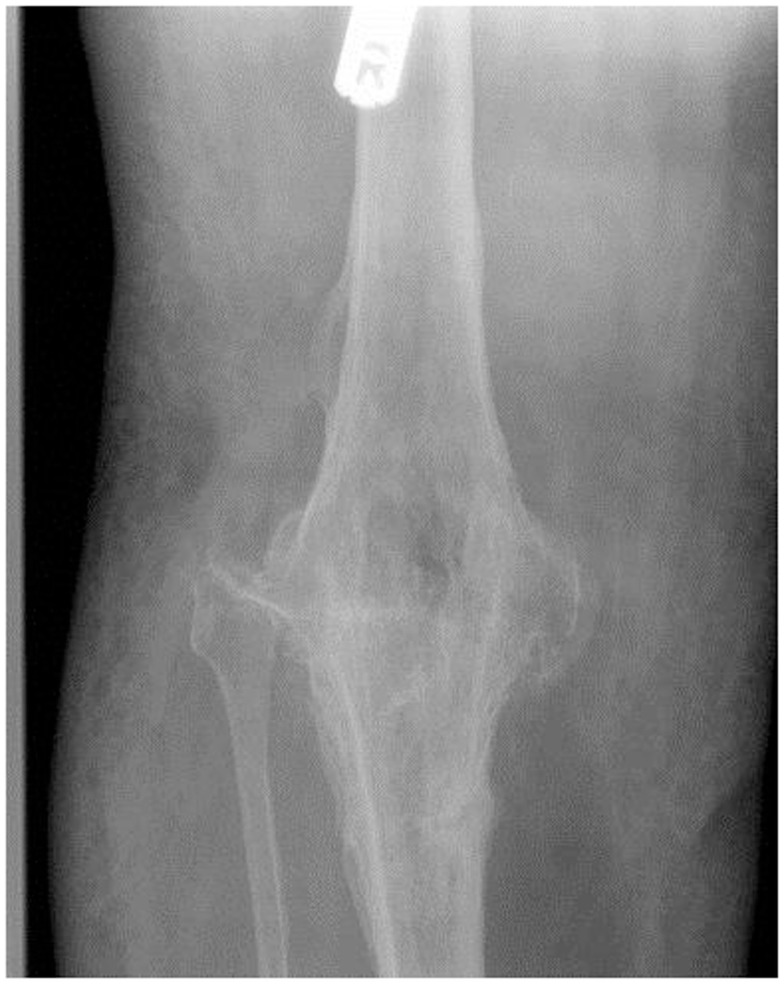
**Complete irrepairable rupture of the tendon resulting in instability, and ultimately knee arthrodesis**.

Four months later, the patient was referred to our unit with persistent deep infection of the knee joint, and overlying skin and soft tissues breakdown. Radiographs demonstrated anterior subluxation of the flat cement spacer disk with its anterior edge lying anterior to the anatomical site of the patella tendon (Figure 1). At our unit, the patient was taken to theater for a further debridement, and intra-operatively noted to have a complete mid-substance rupture of the patella tendon. Following debridement, an interval prosthesis was inserted but the infection persisted. When stable joint reconstruction could not be achieved in view of the patella tendon disruption, a knee arthrodesis (Figure 2) was attempted using a monolateral frame (Orthofix, Verona, Italy). In view of non-union, this was revised to an intramedullary nail arthrodesis using a long nail (Biomet, Bridgend, UK). In view of persistent infection, the arthrodesis nail was removed and intramedullary compartmental debridement performed. Wound healing over the anterior of the knee was eventually achieved by raising a pedicle medial gastrocnemius flap to cover the bony and soft tissue defect.

At final follow-up 24 months post-operatively, the patient had persistent knee pain, and functional assessment revealed an Oxford Knee Score (OKS) of 12 out of 48.

### Case 2

A 78-year-old male underwent a primary right TKA for osteoarthritis. Within 6 weeks of surgery, the patient had an established intra-articular prosthetic knee joint infection. First-stage revision was performed at the referring hospital where the infected knee prostheses were removed, infected soft tissues and bone debrided, and a static cement spacer inserted.

**Figure 3:**
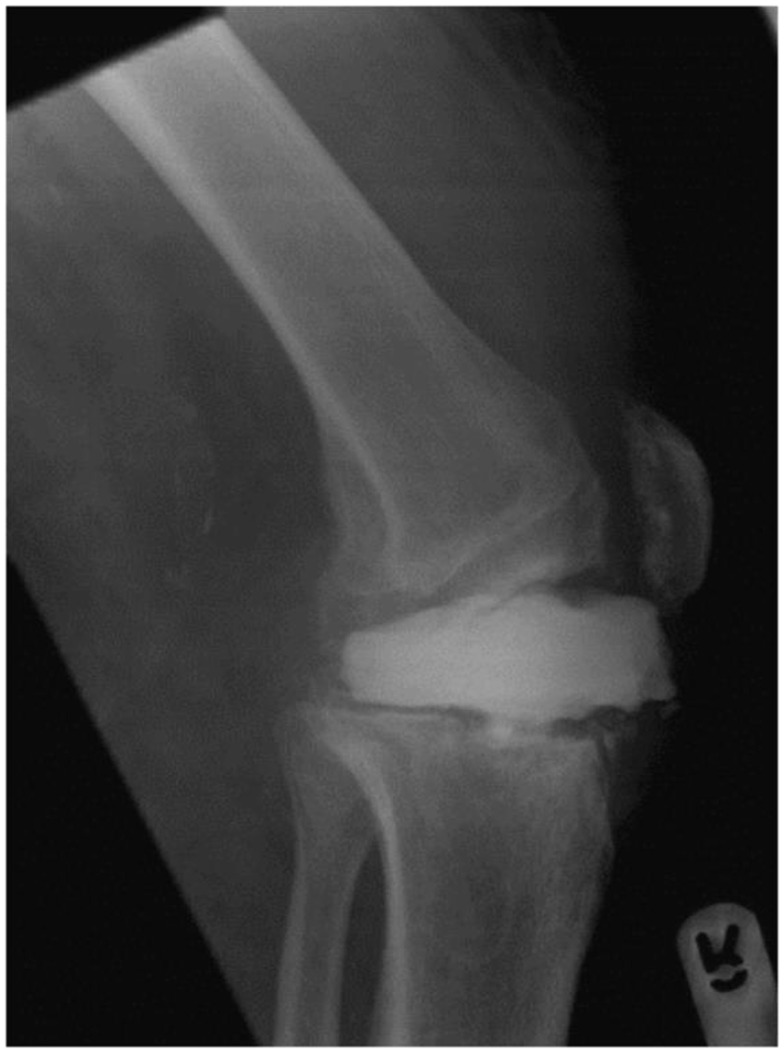
**Partial rupture of the patella tendon in Case 2 demonstrated by the lateral radiographs of the knee showing anterior displacement of the static cement spacer blocks**. Note that the block was not keyed in.

**Figure 4:**
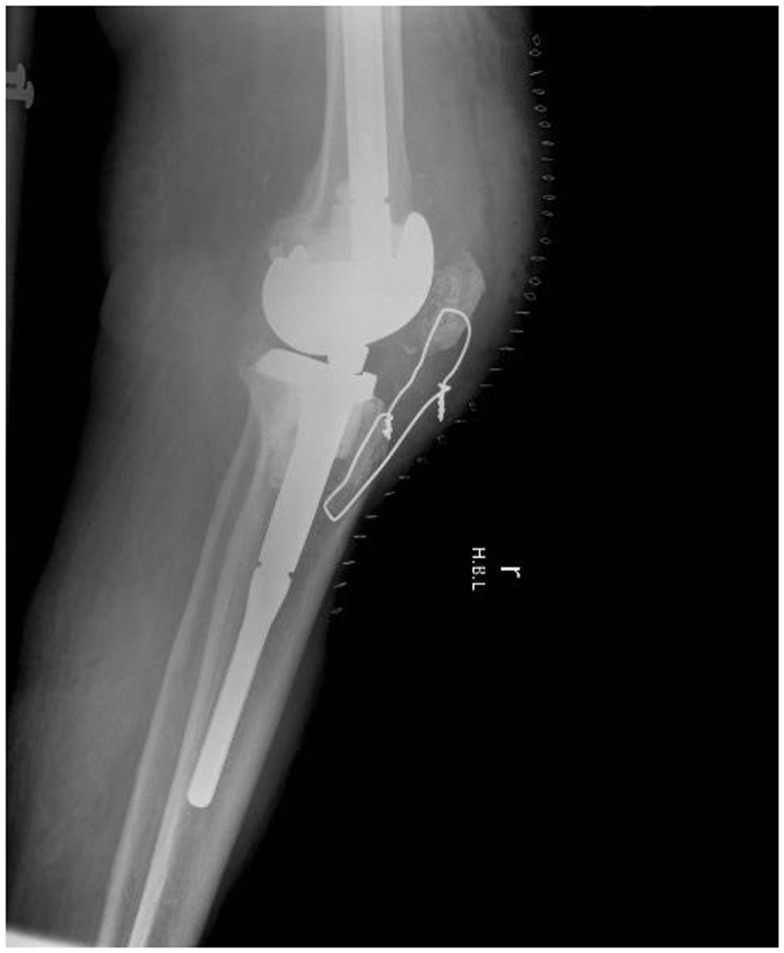
**Lateral radiograph showing a hinged knee prosthesis and a cerclage wire augmenting the patella tendon repair in Case 2**.

Four months later, the patient was referred to our unit with persistent deep knee infection. Radiographs demonstrated that the flat cement spacer disk had subluxed anteriorly and its anterior edge was lying in line with the anatomical site of the patella tendon (Figure 3). At second-stage surgery at our unit, we identified a partial attrition rupture of the patella tendon involving 70% of the tendon. A hinged Noiles knee prosthesis (Depuy, Leeds, UK) was implanted, and the patella tendon was reconstructed using a hamstring autograft supplemented with a cerclage wire to protect the repair (Figure 4). Despite a further washout and debridement, the infection persisted and resulted in loosening of the tibial component requiring a single-stage revision. The patient required two further debridements and the use of vacuum dressing to control the infection. Once the infection settled, a further procedure was performed to enhance the extensor mechanism using a LARS ligament (LARS, Dijon, France).

At final follow-up 24 months following the last surgical procedure, the patient was able to flex the knee to 90° but has an extensor lag of 15° he had an OKS of 18 out of 48. The patient remains on long-term suppressive oral antibiotics.

### Case 3

**Figure 5:**
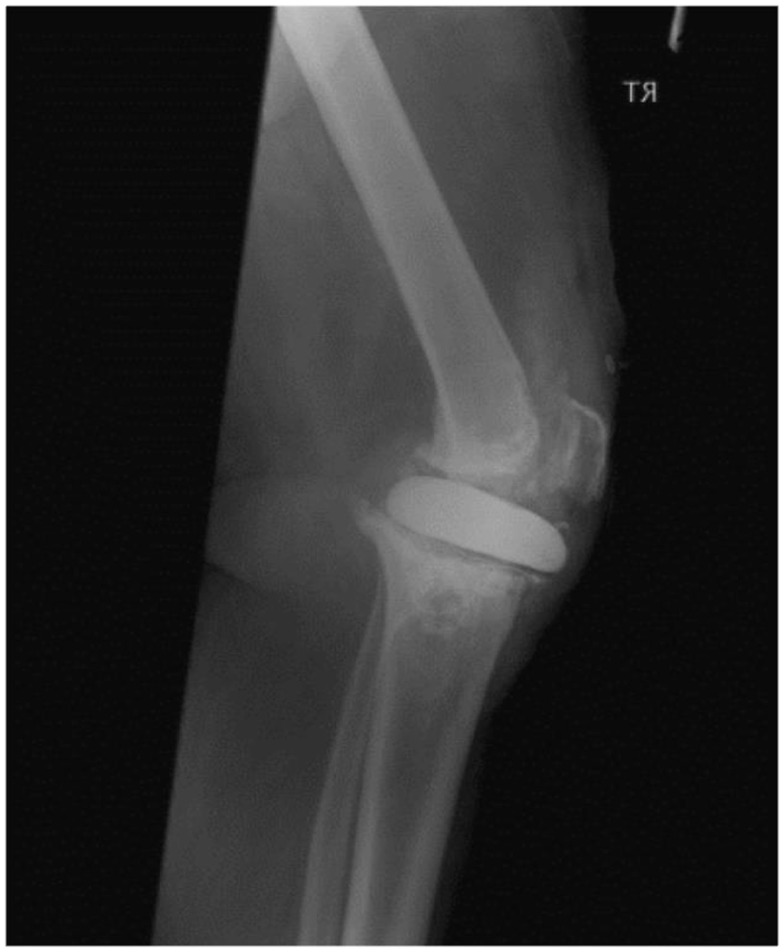
**Partial rupture of the patella tendon in Case 3 demonstrated by the lateral radiographs of the knee showing anterior displacement of the static cement spacer blocks**. Note that the block was not keyed in.

A 70-year-old male underwent a primary right TKA for osteoarthritis. The primary operation was postponed a number of times due to idiopathic septic pustules on the leg carrying coagulase negative *Staphylococci*. After appropriate management of these pustules in consultation with a dermatologist, the primary knee replacement was performed. Following surgery, the patient developed a draining sinus possibly secondary to a stitch abscess in the proximal wound. Cultures grew *Pseudomonas aeroginosa*. This was treated with intravenous antibiotics for 2 weeks followed by oral antibiotics for 6 weeks. The sinus went on to heal and the patient made a good recovery. However, 6 months later, the patient presented again with an acutely swollen knee and radiographs showed loosening of the tibial component. The patient underwent a first-stage revision with insertion of a static cement spacer. The cultures from deep tissues grew coagulase negative *Staphylococcus*. The patient again developed pustules on his leg, delaying his second-stage procedure. Dermatologists advised the second-stage be deferred until the pustules clear up. The patient remained on antibiotics and 18 months later, a lateral radiograph showed that the flat cement spacer disk was eroding the distal femur and had also subluxed anteriorly (Figure 5). The patient was referred to our unit for further management.

At the second-stage revision procedure, a partial attrition rupture of the mid-substance of the patella tendon was found. A hinged Noiles prosthesis was implanted and the patella tendon rupture was reconstructed as described above for Case 2. An displaced femoral shaft fracture just proximal to the tip of the femoral stem was managed with non-weight bearing for 6 weeks and healed well. Three months later, the patient sustained a twisting injury to his knee and re-fractured the femoral shaft. The peri-prosthetic fracture was managed by open reduction and internal fixation using a cable plate system.

At the final follow-up 24 months after the last surgical procedure, the patient remained infection free and independently mobile. His OKS was 20 out of 48. Although the fracture had united, the plate had failed resulting in shortening and malunion at the fracture site.

## Discussion

Although patellar tendon injuries have been previously described with mobile spacers, they have not been described as complications with static spacers. Haddad et al. (7) described patella tendon ruptures with the PROSTALAC functional spacer, and reported a poor outcome but did not provide further detail. This is the first report in the literature describing a series of patella tendon ruptures that resulted from the use of static cement spacer blocks, and their medium term outcome. We have shown that disruption of the extensor mechanism in this vulnerable group of patients leads to significant soft tissue problems that compromise wound healing, knee stability, and mid-term function.

In the management of infected TKAs, there is a shift from using static cement spacer blocks to articulating cement spacers, from intramedullary rod static cement spacers to interval prosthesis, and from two-stage revisions to single-stage revisions. However, the success rate following a two-stage re-implantation in infected TKAs of 85–96% makes it the procedure of choice for most surgeons. The use of antibiotic loaded cement spacers at the first-stage is not without complications though. Static spacers are associated with spacer migration and bone loss (6, 7). Although some studies have shown a higher infection recurrence rates with static cement spacers compared to articulating implants, a recent meta-analyses showed no differences regarding infection control between static and dynamic spacers in the treatment of infected TKA (5). The meta-analyses looked at 25 studies including 318 cases of static spacers and 700 cases of dynamic spacers, but were not able to comment on any differences in complication rates as most studies failed to report them.

Patella tendon ruptures, like deep prosthetic infections, are a catastrophic complication and fortunately uncommon. There have been previous reports of patella tendon injuries following primary TKA and they are known for a poor associated outcome. Many different reconstruction techniques have been described with variable results. In an earlier report by Rand et al. (8), deep infection developed in four out of 17 patients treated for patella tendon ruptures following TKA (8). Their discouraging results prompted them to suggest avoidance of this complication. Jarvela et al. (9) reported an excellent outcome in a case where the patella tendon was reconstructed using a semitendinosus–gracilis graft with an interference screw and staple fixation (9). Mine et al. (10) used a femoral quadriceps tendon for reconstruction and augmented it with a synthetic ligament (10). Their case emphasized that patellar tendon ruptures after TKA should be repaired promptly. Prada et al. (11) in a series of three patients, support the use of allograft including the quadriceps tendon, patella, patella tendon, and tibial tubercle in reconstructing the extensor mechanism, as previously described by Emerson et al. (12). Nazarian et al. (13) achieved a successful clinical outcome for 34 of 40 patients using a distal extensor allograft including fresh frozen tibial tubercle, patella tendon, patella, and quadriceps tendon (13). Two patients from their cohort, however, died and two needed above knee amputations for recurrent infections. Barrack et al. (14) in a series of 14 patients demonstrated the efficacy of using extensor mechanism allografts, either Achilles tendon with calcaneal block or a quadriceps tendon-bone complex, to reconstruct the extensor mechanism (14). Five of their patients, however, had an extensor lag and seven needed aids to mobilize. Although the use of different reconstruction techniques has offered the potential for better clinical outcomes for patients, the results are not always predictable, and significant soft tissue and functional complications remain a problem.

In our series, all injuries occurred in static spacers that were not keyed in to the tibia, and the flat contour increased the likelihood of spacer displacement and tendon injury. In our series, all cases had attrition type tendon ruptures through the mid-substance of the patella tendon and hence surgical repair was not attempted. The presence of deep infection ruled out the possibility of using an allograft to reconstruct the extensor mechanism. The tendon ruptures were reconstructed in the two cases with partial rupture using a semitendinosus autograft. The presence of the patella tendon ruptures significantly complicated the second-stage revision in all the cases. All the cases needed multiple surgeries to control the infection and stabilize the knee. At final follow-up, one of the three patients continues to have residual infection. The combination of this significant complication along with deep infection resulted in a poor functional outcome and significantly increased patient morbidity in all our cases.

The limitation of this case series is that it describes a rare complication. We, however, feel that with an increasing number of knee TKAs and revisions being performed it is important to highlight this devastating but avoidable complication.

## Conclusion

Patella tendon injuries in infected TKAs significantly compromise wound healing, knee stability, and mid-term function. Based on our experience, we advise caution in the use of static cement spacer blocks at first-stage knee revision procedures. There is, however, no evidence in the literature at the moment suggesting a higher complication rate with static spacers. If they are to be used, we recommend that they should be keyed in the bone to prevent patella tendon injuries.

## Conflict of Interest Statement

The authors declare that the research was conducted in the absence of any commercial or financial relationships that could be construed as a potential conflict of interest.
